# Examining the Short-Term Natural History of Developmental Dysplasia of the Hip in Infancy: A Systematic Review

**DOI:** 10.1007/s43465-021-00510-6

**Published:** 2021-09-13

**Authors:** Bryn O. Zomar, Kishore Mulpuri, Emily K. Schaeffer

**Affiliations:** 1 Department of Orthopaedic Surgery, BC Children’s Hospital, 1D.18-4480 Oak Street, Vancouver, BC V6H 3V4 Canada; 2 Department of Orthopaedics, University of British Columbia, Vancouver, BC Canada

**Keywords:** Developmental dysplasia of the hip (DDH), Natural history, Systematic review

## Abstract

**Background:**

This study was an update on the AAOS clinical practice guideline’s analysis of the natural history of developmental dysplasia of the hip (DDH). The objective was to delineate the natural history of clinical instability or radiologic abnormalities of the hip in infants by identifying the proportion of cases that resolved without treatment compared to cases that progressed and/or required treatment.

**Methods:**

We performed a literature search of PUBMED to identify studies which evaluated the natural history of DDH. We used the same search strategy as that utilized in the previous AAOS guidelines, updated to include articles published between September 2013 and May 2021. We assessed the quality of included articles using the Oxford Centre for Evidence-Based Medicine level of evidence and reported study demographics and outcomes using summary statistics.

**Results:**

Twenty-four articles met our eligibility criteria. Most included studies were retrospective (14/24), investigated either the incidence of DDH (8/24) or assessed screening programs (7/24). The most prevalent study population followed were Graf 2A hips (7/24). Most studies were low quality with level of evidence 3 (13/24) or 4 (7/24). Sample sizes ranged from 9 to 3251. Twenty studies reported the number of cases resolved over the follow-up period with a mean rate of 84.3% (95% confidence interval 76.1, 92.6).

**Conclusion:**

We found most mild-to-moderate DDH can resolve without treatment in early infancy, especially in physiologically immature (Graf 2A) hips. More high-quality evidence is needed to properly assess the natural history of DDH as only one included study was a randomized trial.

**Supplementary Information:**

The online version contains supplementary material available at 10.1007/s43465-021-00510-6.

## Background

Developmental dysplasia of the hip (DDH) is a spectrum of hip abnormalities ranging from mild dysplasia in a reduced and stable hip, to a complete and irreducible dislocation of the femoral head from the acetabulum [1]. The broad scope of the DDH severity spectrum has been a major contributor to the lack of widely accepted clinical definitions to provide a basis for comparison of patient populations. Consequently, the true incidence of the condition has been difficult to accurately ascertain. Reported incidence ranges from 1:100 to 1–28:1000 newborns for clinically and/or radiologically detectable hip dislocation receiving an intervention [2, 3]. However, more recent large-scale ultrasound screening studies suggest ultrasound-detectable abnormalities may occur in as many as 5–7% of all newborns [4, 5].

The natural history of DDH has been difficult to clearly delineate due to inconsistent terminology used throughout the literature to describe hip abnormalities, compounded by the spectral nature of the condition. Specifically, recognized abnormalities of the hip in newborns and infants have not been fully characterized and categorized as either pathologic DDH, or self-resolving. This pervasive lack of consistency and reporting, combined with a predominance of single-centre, retrospective studies has limited meaningful cross-study comparison and prevented the generation of high-level evidence on the natural history of the condition.

In September 2014, the American Academy of Orthopaedic Surgeons (AAOS) released a clinical practice guideline (CPG) for the Detection and Nonoperative Management of Pediatric Developmental Dysplasia of the Hip (DDH) in infants up to 6 months of age [6], representing an update on the technical report developed by the AAP in 2000 [7]. During the development of this guideline, the work group prioritized identifying the natural history of clinically unstable or ultrasonographically or radiographically abnormal hips detected in infancy with natural self-correction over time. They identified nine relevant articles and presented summary analyses in the CPG appendix figures [6]. Key findings from the included articles are presented below.

A study conducted by Barlow et al. (1962) examined the early diagnosis and treatment outcomes of a cohort of 9289 newborn babies with Barlow positive (unstable) hips identified by a universal clinical screening program at a single institution in the United Kingdom [8]. The incidence of clinically detected hip instability in this cohort was found to be 16.7 per 1000 at birth. Incidence of instability decreased steadily to reach an incidence of 1.6 per 1000 at 2 months of age in the absence of treatment [6, 8].

Rabin et al. (1965) examined the cross-sectional incidence of radiographic dysplasia in five distinct patient cohorts: < 1 year, 1–2 years, 2–3 years, 3–4 years and > 4 years [9]. Patients were identified from census demographic data collected at an Arizona research centre on the local Navajo population. Incidence rates of radiographic dysplasia were found to be 71.8, 57.1, 0, 0 and 6.8 per 1000 for patients < 1, 1–2, 2–3, 3–4 and > 4 years old, respectively, for a moderately correlated 18.7 per 1000 rate of decrease. Examining this population more broadly, the incidence of radiographic dysplasia or dislocation detected at age 15 months was 32.9 per 1000 while the incidence at 2 years was 7.3 per 1000, reflecting a rate of decrease of 25.6 per 1000 [6, 9]. Schwend et al. re-examined ten patients from this original cohort in 1999 that had remained untreated for acetabular dysplasia throughout the 34-year follow-up period [10]. The mean centre edge angle (CEA) was tracked at 1, 12 and 35 years of age, and was found to increase at a rate of 11.5° over the time interval [6, 10]. Despite overall improvement in hip measurements with maturity, 8/20 hips (5/10 patients) showed subtle but persistent radiographic abnormalities at final follow-up [10].

In 1994, Marks et al. examined whether ultrasound screening for hip instability in neonates could prevent or mitigate late-presenting dislocations [11]. They reviewed a cohort 14,050 newborns referred to a universal screening program at a single institution in the United Kingdom. Infants were sonographically examined at birth, 4 weeks, 9 weeks and 15 weeks, and incidences of sonographic abnormalities at these time points were found to be 60.3, 13.5, 6.1 and 0.1 per 1000, respectively [6, 11]. These findings represent a rate of decrease in sonographic abnormalities of 30.1 per 1000 [6, 11].

In 1999, Bialik et al. reported on a cohort of 9030 infants (18,060 hips) referred to a universal clinical and sonographic screening program at a single institution in Israel [1]. Neonates were examined clinically and by ultrasound at birth, and clinically and by radiograph at 12 months of age. At birth, clinical and/or sonographic abnormalities were detected at a rate of 55.1 per 1000. At 12 months, clinical and/or radiographic abnormalities were detected at a rate of 5 per 1000, representing a rate of decrease of 50.1 per 1000 over this time period [1, 6].

Tegnander et al. (1999) performed a 6–8-year follow-up study of infants with clinically normal but sonographically abnormal hips at birth identified from a cohort of 4973 newborns referred to a universal ultrasound screening program at a single institution in Norway [12]. Infants underwent ultrasound examination at birth and 4–5 months of age, with sonographic abnormalities detected in 34.2 and 2.0 per 1000, respectively. There was no incidence of radiographic abnormality at 6–8 years [6, 12]. In a related study from the same institution, Terjesen et al. (1996) examined incidence of sonographic abnormalities in a cohort of 9952 infants at birth, 2–3 months, 4–5 months and a later unspecified time point [13]. Consistent with Tegnander et al., the authors found an initial incidence of 31.0 per 1000 at birth, decreasing to 2.8 per 1000 at 4–5 months and 1.6 at the further follow-up time [13].

In a randomized controlled trial, Wood et al. (2000) examined the impact of abduction splintage on clinically stable but sonographically dysplastic hips, as measured by acetabular coverage at 2–6 weeks of age (trial start) and 3–4 months of age (trial end) [14]. A total of 63 hips in 44 infants were randomized to abduction splintage or observation for a period of 3 months. The observed cohort (18 hips) were examined to provide insight on the natural history of acetabular coverage, which was found to increase from 36.7% at birth to 48.6% at 3 months in the absence of any treatment. While improvement in acetabular coverage was significantly better in the splinted group (32.8–54.3%), there was no appreciable difference in the acetabular index between the two groups as measured on plain radiograph at 2 years of age [6, 14].

Another prospective study by Castelein et al. (1992) followed 144 clinically normal but sonographically abnormal newborn hips without treatment for a mean of 8 months, identified from a cohort of 691 clinically normal hips [15]. The rate of sonographic abnormality decreased from 208.4 per 1000 at birth to 10.1 per 1000 at 8 months of age [6, 15].

As evidenced in the natural history studies described in the CPG, inconsistent terminology, lack of clarity in reporting, diverse observation periods and variable outcome measures have prevented the generation of strong evidence to provide insight on the natural history of this condition. However, taken together, most cases of either clinical hip instability or sonographic abnormality in the neonate resolved spontaneously during early infancy.

This systematic review presents an update on AAOS CPG guideline’s analysis of the natural history of DDH [6], identifying and analyzing studies published after guideline release. The objective of this systematic review was to delineate the natural history of clinical instability or radiologic abnormalities of the hip in infants by identifying the proportion of cases that resolved without treatment compared to cases that progressed and/or required treatment.

## Methods

### Search Strategy

We performed a literature search of PUBMED to identify studies for inclusion in our review of the natural history of DDH. We used the same search strategy as that utilized in the AAOS guidelines for the Detection and nonoperative management of pediatric developmental dysplasia of the hip in infants up to 6 months of age published in 2014 (Supplementary Material 1) [6] We updated the search strategy to identify articles published between September 9, 2013 (the end date of included studies in the AAOS guidelines) and May 19, 2021, when the search was conducted. Briefly, mesh headings consisted of: “Hip Dislocation” (with and without congenital), “hip” or “hip joint” or “femur head” and “joint instability” or “bone diseases, developmental”, and combined with “infant” or “child, preschool”. Title and keywords included, but were not limited to: “hip(s)”, “dysplasia”, “dysplastic”, “dislocat*” “subluxat*”, “unstable”, “instability”, “screening”, “ultrasound”, “developmental”, or “congenital”. The search was date-limited and restricted to English language, original clinical human studies.

### Eligibility Criteria

To be consistent with the AAOS work group, we based our study selection criteria on those published in the AAOS guidelines [6]. To be included, articles must be of DDH, a full report of a clinical study, appear in a peer-reviewed publication, published in English, and include humans. Additionally, studies must include untreated patients who have at least one follow-up time point described. Studies were excluded if they were an in vitro or biomechanical study, were performed on cadavers, included less than ten patients per group, or described only treated patients. Studies were also excluded if the results were not presented quantitatively, if there was no follow-up or if there was < 50% follow-up for any given follow-up time point, if the study was a retrospective non-comparative case series, medical records review, meeting abstract, historical article, editorial, letter or commentary. Case series with non-consecutive enrollment of patients were also excluded. To update the search from the previous AAOS guidelines, we only included studies published between September 9, 2013 and May 19, 2021.

### Abstract and Full-Text Screening

Two reviewers independently screened all titles and abstracts of studies identified from the literature search. The reviewers determined whether each study should be included for full-text review or excluded based on the eligibility criteria. The same reviewers pulled the full text for included articles and reviewed to determine if each still met the eligibility criteria. At both title and abstract review and full text review, consensus discussion was held to resolve any cases of disagreement. Reference lists of included articles were searched for any additional relevant studies.

### Data Extraction

An excel spreadsheet was created and used by the reviewers to extract data from each included full text article. The following data points were collected for each article: (1) study title; (2) authors; (3) publication year; (4) study design; (5) age of inclusion for patients; (6) total number of patients included; (7) number of untreated patients included (natural history); (8) any comparator groups; (9) total length of follow-up; (10) assessment time points; (11) how diagnosis was assessed (i.e. clinical exam, ultrasound, X-ray, or a combination); (12) included diagnoses; (13) outcomes assessed; (14) outcome results at each time point; (15) outcome results at presentation; (16) number of cases that resolved; (17) number of cases that progressed; (18) whether loss to follow-up was reported; (19) number or percent of cases lost to follow-up. The first three articles had data independently extracted by both reviewers (B.O.Z. and E.K.S) and after discussion and consensus on data collection process and procedures, the remaining articles were split between the reviewers. Any unclear data points were discussed and clarified with both reviewers.

### Quality Assessment

Study heterogeneity and the predominance of retrospective cohort studies included in our review prevented a formal risk of bias assessment. However, the level of evidence of all included full text was assessed using the Oxford Centre for Evidence-Based Medicine 2011 Levels of Evidence [16]. Two reviewers independently assessed all articles and discrepancies in rating were resolved through discussion.

### Analysis

We were unable to perform a meta-analysis for this study as the reporting of trial results was inconsistent. We summarized our results using descriptive statistics. We used frequencies and proportions to present the study characteristics and reported outcomes.

## Results

**Fig. 1:**
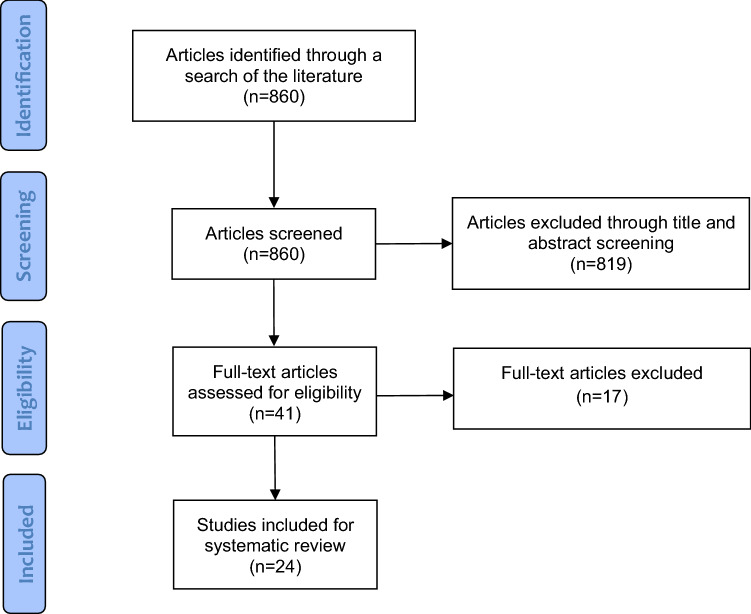
Reporting items for systematic reviews and meta-analyses (PRISMA) study flow diagram

Our literature search resulted in 860 articles, of which 24 met our eligibility criteria for inclusion in the study [17–40]. The search and selection process is outlined in Fig. 1. A 2018 review on the natural history of DDH was excluded based on our inclusion criteria, but a search of the reference list did not identify any additional potentially relevant articles [41]. Demographic features of the studies are summarized in Table 1. Included in our review were 9 prospective cohort studies, 14 retrospective studies and 1 randomized controlled trial. Most of the studies investigated the incidence of DDH (8/24) or assessed screening programs (7/24). A range of follow-up periods were reported across the studies, though almost all covered a period of at least three months (16/24). The most prevalent study population were Graf type 2A hips (7/24), and the age of inclusion ranged from newborn to 7 months.

**Table 1:** Demographic characteristics of included studies

Title	First author	Year	Study type	Age of inclusion	Length of follow-up for natural history	Included diagnoses for natural history	OCEBM question type
Clinical diagnosis only							
The ‘clicky hip’: to refer or not to refer?	Humphry S [17]	2018	Prospective	0–7 months	NR	‘Clicky’ hips	Incidence
Correlations between ultrasonographic and subsequent radiographic findings of developmental dysplasia of the hips	Tan SHS [18]	2019	Retrospective	Newborn	1 year	Positive Barlow, positive Ortolani or clicking hips	Diagnosis
Pavlik harness initiation on Barlow positive hips: can we wait?	Cook KA [19]	2019	Retrospective	0–2 weeks	12 weeks	Barlow positive	Prognosis
US diagnosis only							
Orthopedic and ultrasound assessment of hip stability of newborns referred by pediatricians with suspected developmental dysplasia	Cruz MAF [20]	2020	Retrospective	Newborn	Up to 8 weeks	Borderline/doubtful US	Incidence
Orthopedic assessment of the hips in newborns after initial pediatric survey	Gonzalez FC [21]	2019	Prospective	Newborn	6 months	Graf 1 and 2A	Incidence
Incidence and follow-up outcomes of developmental hip dysplasia of newborns in the Western Mediterranean Region	Çekiç B [22]	2015	Prospective	> 6 weeks	12 weeks	Graf 2A	Incidence
A new measurement method in Graf technique: prediction of future acetabular development is possible in physiologically immature hips	Yavuz OY [23]	2014	Retrospective	< 2 months	3 months	Graf 2A	Diagnosis
Incidence and treatment of developmental hip dysplasia in Mongolia: a prospective cohort study	Munkhuu B [24]	2013	Prospective	Newborn	Variable (monthly until mature)	Graf 2A	Incidence
Results of a universal ultrasonographic hip screening program at a single institution	Güler O [25]	2016	Retrospective	1 month	3 months	Graf 2A	Screening
Ultrasonographic Graf type IIa hip needs more consideration in newborn girls	Omeroğlu H [26]	2013	Retrospective	3–4 weeks	12 weeks	Graf 2A	Prognosis
Introducing universal ultrasound screening for developmental dysplasia of the hip doubled the treatment rate	Olsen SF [27]	2018	Retrospective	Newborn	4–12 weeks	Physiologically immature (alpha angle 50–60 degrees)	Screening
Universal versus selective ultrasound screening for developmental dysplasia of the hip: a single-centre retrospective cohort study	Westacott DJ [28]	2018	Retrospective	0–6 weeks universally screened; 8 weeks selectively screened	> 2 years	All	Screening
Optimizing the time for developmental dysplasia of the hip screening: earlier or later?	Gokharman FD [29]	2019	Prospective	< 6 months	12 weeks	All Graf	Screening
Radiographic follow-up of DDH in infants: are X-rays necessary after a normalized ultrasound?	Sarkissian EJ [30]	2015	Retrospective	< 8 weeks	12 months	Abnormal US (laxity, instability, or alpha angle < 60)	Incidence
Abduction treatment in stable hip dysplasia does not alter the acetabular growth: results of a randomized clinical trial	Pollet V [31]	2020	Randomized Controlled Trial	3–4 months	12 weeks	Graf 2B and 2C	Treatment Benefits
Early neonatal universal ultrasound screening for developmental dysplasia of the hip: a single institution observational study	Treiber M [32]	2021	Prospective	Newborn	2–12 weeks	Graf 2A, 2B, 2C, D, 3A	Screening
Clinical and US diagnosis							
Clicky hip alone is not a true risk factor for developmental dysplasia of the hip	Nie K [33]	2017	Prospective	Mean age 13.8 weeks	NR	‘Clicky’ hips, Graf 2A	Incidence
An index for diagnosing infant hip dysplasia using 3-D ultrasound: the acetabular contact angle	Mabee MG [34]	2016	Prospective	Range 4–183 days	> 3 months	Hip laxity, asymmetrical skin creases or risk factors	Diagnosis
Treatment patterns and outcomes of stable hips in infants with ultrasonic dysplasia	Kim HKW [35]	2019	Prospective	0–3 months	> 3 months	Negative Barlow and Ortolani, alpha angle between 40 and 55 degrees and FHC between 10 and50%	Treatment Benefits
Is there a predilection for breech infants to demonstrate spontaneous stabilization of DDH instability?	Sarkissian EJ [36]	2014	Retrospective	< 8 weeks	4–18 weeks	Clinically stable, hip laxity on dynamic US and no history of treatment	Prognosis
Natural history of hip instability in infants (without subluxation or dislocation): a three year follow-up	Pruszczynski B [37]	2014	Retrospective	< 2 months	12–228 weeks	Joint instability under stress but reduced at rest	Prognosis
Selective ultrasound screening for developmental hip dysplasia: effect on management and late detected cases. A prospective survey during 1991–2006	Laborie LB [38]	2014	Retrospective	Newborn	> 6 weeks	Clinically or sonographically unstable but not dislocatable, or mild sonographic dysplasia	Screening
Acetabular dysplasia at the age of 1 year in children with neonatal instability of the hip	Wenger D [39]	2013	Retrospective	Newborn	1 year	All	Incidence
Risk factor assessment and a ten-year experience of ddh screening in a well-child population	Kural B [40]	2019	Retrospective Case–Control	1 month	18 months	Unclear	Screening

*NR* not reported, *US* ultrasound, *FHC* femoral head coverage, *OCEBM* Oxford Centre for Evidence-Based Medicine

**Fig. 2:**
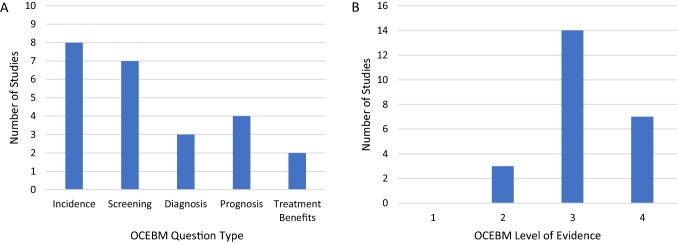
Frequency of studies **A** addressing each question type and **B** evaluated for level of evidence according to the Oxford Centre for Evidence-Based Medicine (OCEBM)

Reported results and study characteristics are summarized in Table 2. The sample size for patients followed for natural history was variable across the studies ranging from 9 to 3251 patients. There was inconsistent reporting of sample size across the studies with some reporting the number of patients, others reporting the number of hips, or reporting both. In total, 7606 patients were followed across 20/24 included studies that specifically reported patient number. Three of the remaining 4 studies reported the number of hips followed, totaling 1357. Overall quality of the studies was low, with almost all studies were rated as a three or four for level of evidence (13/24 and 7/24, respectively; Fig. 2). The most reported outcome assessment among the studies was the Graf classification (15/24). Twenty studies reported the number of patients whose DDH either resolved without treatment or progressed and/or required treatment during follow-up. Of these studies, the rate of DDH resolving without treatment ranged from 40 to 100%, with a mean of 84.3% [95% confidence interval (76.1, 92.6)]. Four studies reported 100% of patients had their DDH resolve during follow-up. We were able to determine loss to follow-up, or it was reported, in 13 studies and ranged from 0 to 35.7%.

**Table 2:** Study characteristics extracted and evaluated from included articles

Title	First author	Total number of patients (hips)	Number of patients (hips) followed for natural history	Number of patients (hips) resolved*, [%]	Number of patients (hips) progressed and/or treated*, [%]	Loss to follow-up for natural history	Outcomes assessed	Level of evidence
Clinical diagnosis only								
The ‘clicky hip’: to refer or not to refer?	Humphry S [17]	69	19	8 [42%]	11 [58%]	NR	–Graf classification	4
Correlations between ultrasonographic and subsequent radiographic findings of developmental dysplasia of the hips	Tan SHS [18]	160	160	NR	NR	NR	–Graf classification –Harcke’s dynamic US screening –Terjesen’s femoral head coverage	2
Pavlik harness initiation on barlow positive hips: can we wait?	Cook KA [19]	30 (39)	30 (39)	12 [40%] (17 [44%])	18 [60%] (22 [56%])	0%	–Acetabular index	4
US diagnosis only								
Orthopedic and ultrasound assessment of hip stability of newborns referred by pediatricians with suspected developmental dysplasia	Cruz MAF [20]	448	26	24 [92%]	NR	7.7%	–Graf classification –clinical signs	3
Orthopedic assessment of the hips in newborns after initial pediatric survey	Gonzalez FC [21]	34 (68)	32	NR	NR	NR	–Graf classification	3
Incidence and follow-up outcomes of developmental hip dysplasia of newborns in the Western Mediterranean Region	Çekiç B [22]	1162 (2324)	257	191[74%]	10 [4%]	21.7%	–Graf classification	3
A new measurement method in Graf technique: prediction of future acetabular development is possible in physiologically immature hips	Yavuz OY [23]	NR (1391)	NR (314)	NR (279 [89%])	NR (35 [11%])	NR	–Alpha, beta and gamma angles	2
Incidence and treatment of developmental hip dysplasia in Mongolia: a prospective cohort study	Munkhuu B [24]	8356 (16,712)	1146 (1715)	607 [53%]	149 [13%]	30%	–Graf classification –treatment	3
Results of a universal ultrasonographic hip screening program at a single institution	Güler O [25]	4782 (9564)	463 (737)	353 [76%] (562 [76%])	22 [5%] (25 [3%])	19%	–Graf classification –Risk factors	3
Ultrasonographic Graf type IIa hip needs more consideration in newborn girls	Omeroğlu H [26]	321 (431)	321 (431)	185 [58%] (249 [58%])	29 [9%] (36 [8%])	33.9%	–Graf classification	3
Introducing universal ultrasound screening for developmental dysplasia of the hip doubled the treatment rate	Olsen SF [27]	4245	459	432 [94%]	27 [6%]	NR	–Graf classification –Risk factors	4
Universal versus selective ultrasound screening for developmental dysplasia of the hip: a single-centre retrospective cohort study	Westacott DJ [28]	10,015 universally screened, 18,053 selectively screened	NR	NR	NR	NR	–Delayed diagnosis –Treatment	3
Optimizing the time for developmental dysplasia of the hip screening: earlier or later?	Gokharman FD [29]	1010 (2020)	1010 (2020; 1012 followed)^b^	NR (154 [21%] between 4–12 weeks; 214 [13%] between 8–12 weeks)	NR (49 [7%] between 4–12 weeks; 141 [9%] between 8–12 weeks)	360 followed at 4 and 12 weeks, 819 followed at 8 and 12 weeks	–Graf classification	3
Radiographic follow-up of DDH in infants: are X-rays necessary after a normalized ultrasound?	Sarkissian EJ [30]	115	36	22 [61%]	14 [39%]	NR	–Graf classification –Acetabular index	4
Abduction treatment in stable hip dysplasia does not alter the acetabular growth: results of a randomized clinical trial	Pollet V [31]	104	49	39 [80%]	10 [20%]	0%	–Graf classification –Alpha angle –Acetabular index –Tonnis classification	2
Early neonatal universal ultrasound screening for developmental dysplasia of the hip: a single institution observational study	Treiber M [32]	21,676 (43,352; 10,979 followed)	NR (1001)	NR (851 [85%]^a^)	NR (2 [0.2%])	11.7%	–Graf classification	3
Clinical and US diagnosis								
Clicky hip alone is not a true risk factor for developmental dysplasia of the hip	Nie K [33]	362	9	9 [100%]	0	NR	–Barlow –Ortolani –Graf classification –Harcke	4
An index for diagnosing infant hip dysplasia using 3-D ultrasound: the acetabular contact angle	Mabee MG [34]	85 (114)	23 (34)	23 [100%] (34 [100%])	0	NR	–Alpha angle –Acetabular contact angle	3
Treatment patterns and outcomes of stable hips in infants with ultrasonic dysplasia	Kim HKW [35]	80 (107)	NR (42)	NR (25 [60%])	NR (2 [5%])	35.7%	–Alpha angle –Percent FHC –Acetabular index	3
Is there a predilection for breech infants to demonstrate spontaneous stabilization of DDH instability?	Sarkissian EJ [36]	79 (122)	79 (122)	NR (81 [66%])	NR (41 [34%])	NR	–Graf classification –Treatment –Risk factors	3
Natural history of hip instability in infants (without subluxation or dislocation): a 3 year follow-up	Pruszczynski B [37]	25 (48)	25 (48)	25 [100%] (48 [100%])	0	0	–Many US measurements	4
Selective ultrasound screening for developmental hip dysplasia: effect on management and late detected cases. A prospective survey during 1991–2006	Laborie LB [38]	11,190	3251	2700 [83%]	551 [17%]	3%	–Clinical stability –US alpha angle, stability, position of femoral head –X–ray acetabular index, position of femoral head	3
Acetabular dysplasia at the age of 1 year in children with neonatal instability of the hip	Wenger D [39]	332	174	NR	NR	27%	–Acetabular index –Signs of AVN	3
Risk factor assessment and a 10-year experience of DDH screening in a well-child population	Kural B [40]	57 (97)	37	37 [100%]	0	NR	–Risk factors	4

*NR* not reported, *US* ultrasound, *AVN* avascular necrosis

*The number resolved and progressed/treated may not equal the number followed for natural history due to loss to follow-up or poor reporting

^a^Unclear or not reported number that were treated

^b^Graf type 1 were included in the total natural history sample followed, but were not included in the number resolved or progressed/treated

## Discussion

The true natural history of DDH has been difficult to ascertain, in part because much of the existing evidence in the historical literature is from retrospective or single-centre studies. Additionally, the wide severity spectrum encompassed by the disorder has led to confusion in diagnostic terminology, as well as inconsistencies in treatment and management [42]. Further hindering the study of the natural history, it is well recognized that when left undetected or untreated, DDH can lead to debilitating complications later in life [43, 44].

There is consensus that early diagnosis is critical to optimize outcomes and mitigate long-term disability for children. However, there is also concern for the potential to overtreat, particularly with universal ultrasound screening in newborn infants. During the development of the AAOS clinical practice guidelines on DDH [6], a comprehensive review of the literature found that the natural history of DDH largely appears to depend where the pathology lies on the DDH severity spectrum, with mild dysplasia often resolving without any clinical symptoms during childhood. Most natural history studies summarized in the AAOS review found the majority of DDH cases discovered by clinical examination or ultrasound study in newborns represented hip laxity or immaturity, rather than pathological DDH [6]. Specifically, their analysis revealed 60–80% of clinically identified abnormalities and 90% of ultrasonographic abnormalities resolved in early infancy without treatment. However, these findings are not likely to be as reflective in more severe cases of dislocation.

In 2018, Sakkers et al. reviewed the natural history of abnormal hip ultrasound findings in infants under 6 months of age [41]. The authors reviewed and analyzed 13 561 hips and concluded that for Graf 2A to 2C hips, 80–97% normalized without treatment, likewise in more than 50% of Graf 3 hips. In contrast, less than 50% of Graf 4 hips normalized without treatment. The study concluded that the natural history of DDH is relatively benign in well-centered hips [41].

In this context, consideration must be given to potential overtreatment of infants, particularly in more mild cases of hip instability or radiological dysplasia. Brace treatment is common in these cases; however, it is unclear whether this approach provides significant benefit above careful observation by ultrasound. While a conservative, less costly approach, brace treatment is not without potential complications and drawbacks. There are still substantial healthcare costs and resources associated with brace treatment but there is also an underrecognized psychosocial cost regarding prevention or disruption of mother–infant bonding in the newborn period [45–47]. Coping with the difficulties of brace treatment can be stressful for families, particularly mothers of newborns, but the ultimate psychosocial impact has been under-researched to date. A recent survey study on the experiences of patients and caregivers during care for DDH revealed the challenges bracing can impose on daily life and highlighted the need to take the patient experience into consideration [48]. A more complete understanding of the natural history of DDH can allow for the avoidance of unnecessary treatment, potentially decreasing both the psychosocial impact of disrupted mother–infant bonding and needed healthcare resources and costs.

This systematic review serves to update the review performed during the development of the AAOS guidelines [6], examining studies published after their September 9, 2013 search date end point. Several of the historical studies included in the AAOS review examined DDH natural history by the incidence of either clinical or radiologic hip abnormalities at advancing age throughout infancy and childhood [1, 9, 11, 12]. To delineate the course of DDH natural history more specifically, our review only included studies that had some extent of follow-up data on the study population. We also expanded upon the Sakkers et al. [41] criteria, not limiting our search to ultrasound findings or infants under 6 months of age. It was possible to ascertain the rate of spontaneous resolution in 20/24 studies. The mean rate of spontaneous resolution was 84.3%. This finding is generally consistent with those of the AAOS review and Sakkers et al., whereby most hips appeared to resolve without treatment during infant development [6, 41]. However, it is important to consider that many of these cases of resolution likely occur in milder forms of DDH pathology, and severe forms most probably require intervention. Given the differences in study populations, outcome measures and discrepant results reporting, resolution rates are difficult to compare or combine across the severity spectrum. For example, seven of the included studies examined only Graf 2A hips in their natural history population [22–27, 33]. Graf 2A hips are typically recognized as physiologically immature hips, not necessarily pathologically DDH hips. In contrast, other studies included more severe Graf types [28, 29, 31, 32, 39], and/or evidence of clinical instability or a positive Barlow test [18, 19, 36–38]. Consequently, these results must be interpreted with caution to avoid undertreatment of potentially pathologic hips. Indeed, Cook et al. reported only a 43.6% spontaneous resolution rate in Barlow positive hips, with 17 hips in 12 patients normalizing without treatment from a population of 39 hips in 30 patients [19].

This systematic review has several limitations. First, the search was not performed across an exhaustive list of databases. However, the search strategy was comprehensive and hand searches of reference lists of multiple included articles as well as the Sakkers review [41] did not result in any additional potential studies for inclusion. Despite our inclusive search strategy, almost all articles included in our study only evaluated the short-term natural history of DDH in early infancy. Second, we only included published, peer-reviewed articles available in English, potentially omitting relevant non-English language studies, theses or conference proceedings. However, this was consistent with that of the AAOS work group review [6].

This review was also limited by the evidence included in the review. Study heterogeneity prevented meta-analysis or synthesis of results. This heterogeneity was apparent in study design and question type, as well as patient population, included diagnoses, length of follow-up and follow-up intervals. Lack of clarity in reporting across studies also prevented comprehensive meta-analysis or results synthesis. Several studies did not report key outcomes, or presented aggregate results of natural history and treated patients. Finally, assessment of study quality revealed a predominance of level 3 and 4 evidence (87.5%, Fig. 2), as assessed by the Oxford Centre for Evidence-based Medicine 2011 Levels of Evidence [16]. With no level 1 studies and only three level 2 studies included in this review, there is an evident need for more prospective, appropriately powered randomized controlled trials or comparative effectiveness studies.

Overall, this systematic review update on the natural history of DDH revealed that most mild-to-moderate DDH can resolve without treatment in early infancy. This may especially be the case in physiologically immature (Graf 2A) or radiologically dysplastic hips. High level evidence generated by prospective studies will be required to fully understand which hips are safe to monitor without treatment. Non-inferiority randomized controlled trials are a particularly well-suited design to answer these questions and should be a consideration in future research.

## Supplementary Information

Below is the link to the electronic supplementary material.

Supplementary file1 (DOCX 14 KB)
