# Supplementary material for: Examining the Short-Term Natural History of Developmental Dysplasia of the Hip in Infancy: A Systematic Review
Source: Indian J Orthop. 2021 Sep 13;55(6):1372–87. doi: 10.1007/s43465-021-00510-6 (PMC8688652; doi:10.1007/s43465-021-00510-6)
Supplement: Supplementary file 1 — Supplementary file1 (DOCX 14 KB) [file 43465_2021_510_MOESM1_ESM.docx]

Title: Examining the Natural History of Developmental Dysplasia of the Hip: A Systematic Review

Journal: Indian Journal of Orthopaedics

Authors: Bryn O Zomar, Kishore Mulpuri, Emily K Schaeffer

Corresponding Author Affiliation: BC Children’s Hospital, Vancouver, BC, Canada

Corresponding Author Email: Emily.Schaeffer@cw.bc.ca

**Online Resource 1**

Literature Search Strategy

#1

"Hip Dislocation, Congenital"[mh]

#2

Hip Dislocation[mh]

#3

hip[mh] OR hip joint[mh] OR femur head[mh]

#4

Joint Instability[mh] OR "Bone Diseases, Developmental"[mh:noexp]

#5

#3 AND #4

#6

#1 OR #2 OR #5

#7

Infant[mh] OR "Child, preschool"[mh]

#8

#6 AND #7

#9

hip[titl] OR hips[titl]

#10

dysplasia[tiab] OR dysplastic[tiab] OR dislocat*[tiab] OR luxation[tiab] OR subluxat*[tiab] OR instability[tiab] OR unstable[tiab] OR stability[tiab] OR abnormal*[tiab]

#11

screening[tiab] OR ultrasound[tw] OR exam[tiab] OR examination[tw]

#12

congenital[tw] OR developmental[tw]

#13

infan*[tw] OR newborn*[tw] OR babies[tiab] OR neonatal[tiab] OR pediatric[tiab] OR paediatric[tiab] OR early[titl]

#14

#9 AND #10 AND (#11 OR #12) AND (#7 OR #13)

#15

epidemiolog*[tw] OR incidence[tw] OR prevalence[mh] OR Risk factors[mh]

#16

#15 AND (#1 OR (#2 AND #12))

#17

hip/abnormalities[ot]

#18

#17 AND (#12 OR #13)

#19

#8 OR #14 OR #16 OR #18

#20

("2021/05/19"[Date - Entrez] : "2013/09/16"[Date - Entrez]) AND English[lang]

#21

(animal[mh] NOT human[mh]) OR veterinary[sh] OR cadaver[mh] OR cadaver*[titl] OR ((comment[pt] OR editorial[pt] OR letter[pt] OR "historical article"[pt]) NOT "clinical trial"[pt]) OR addresses[pt] OR news[pt] OR "newspaper article"[pt] OR pmcbook

#22

#19 AND #20 NOT #21
